# Extracellular Vesicle microRNAs in the Crosstalk Between Cancer Cells and Natural Killer (NK) Cells

**DOI:** 10.3390/cells14211697

**Published:** 2025-10-29

**Authors:** Nicolo Toldo, Yunjie Wu, Muller Fabbri

**Affiliations:** Center for Cancer and Immunology Research, Children’s National Hospital, Washington, DC 20010, USA; nicolo.toldo@childrensnational.org (N.T.); ywu3@childrensnational.org (Y.W.)

**Keywords:** extracellular vesicles, exosomes, microRNAs, Natural Killer cells, cancer, tumor microenvironment, immunotherapy

## Abstract

The term extracellular vesicles (EVs) includes a variety of anucleated, non-self-replicative particles released by cells, whose cargo content is compartmentalized by a lipidic bilayer membrane and includes proteins, DNA, and RNA (both coding and non-coding) molecules. MicroRNAs (miRs) are small non-coding RNA involved in gene expression regulation that functionally participate in inter-cellular communication as EV cargo. Natural Killer (NK) cells are innate immunity lymphocytes specialized in the killing of cancer cells and virally infected cells. Increasing evidence shows that NK cell-derived EVs contribute to the anti-tumoral activity of NK cells and that such effects are, at least in part, mediated by the miR cargo of these EVs. Conversely, cancer cells release EVs whose cargo includes proteins and miRs that impair NK cell function. These interactions highlight a central role for EV miRs both in the NK-mediated cytotoxicity and as a major immune-escape mechanism for cancer cells, ultimately contributing to the overall success or failure of NK cells in eliciting their anti-tumoral activity.

## 1. Introduction

Natural Killer (NK) cells are CD3-negative and CD56-positive lymphocytes specialized in the killing of cancer cells and virally infected cells. They belong to the innate immunity branch of our immune system since they do not require specific antigen recognition to perform their cytotoxic activity. An increasing number of studies shows that NK cells kill cancer cells not only through “traditional” mechanisms (which include granule exocytosis pathway and death receptor-mediated killing) but also through the transfer of tumor suppressor microRNAs (miRs) as cargo of NK cell-derived extracellular vesicles (EVs) to the cancer cells. Conversely, cancer cells can impair NK cell-mediated cytotoxicity by shuttling specific miRs as the cargo of their EVs to NK cells. After a brief description of NK cells, EVs, and miRs, we will discuss the state-of-the-art literature on this EV-miR-mediated two-way communication between NK cells and cancer cells and their emerging implications for cancer immunology and therapy.

## 2. Natural Killer Cells, Extracellular Vesicles, and microRNAs

### 2.1. Natural Killer (NK) Cell Classification, Maturation, and Cytotoxicity Mechanisms

NK cells represent 5–10% of all circulating lymphocytes in human blood [1]. They secrete multiple different types of cytokines but mostly interferon-γ (IFNγ), therefore significantly contributing to the orchestration of adaptive immune responses [2]. They derive from a common lymphoid progenitor in the bone marrow that renews about every 2 weeks on average [3], and their function requires the transcription factor TBX21 and EOMES. Currently, they are considered a subset of the Innate Lymphoid Cell (ILC) family, which also includes ILC1 cells (requiring TBX21 transcription factor and secreting IFNγ and TNFα and involved in Type 1 immunity with an unclear role in cancer), ILC2 cells (requiring GATA3 transcription factor and secreting IL-4, IL-5, IL-9, and IL-13 to promote a Type 2 immunity with an unclear role in cancer), ILC3 cells (requiring RORγt transcription factor and secreting IL-17 and IL-22 to promote a Type 3 immunity, which can be pro- or anti-tumoral depending on context), and lymphoid tissue-inducer (LTi) cells (requiring the RORγt transcription factor and secreting RANK, lymphotoxin (LTA), TNFα, and IL-17 to promote lymphoid organogenesis) [4]. In order to avoid the destruction of healthy cells, NK cells undergo an “educational” process that involves the interaction between their immunoreceptor tyrosine-based inhibitory motifs (ITIMs) and class I of the Major Histocompatibility Complex (MHC-I) [5,6]. NK cell survival, development, and activation depends on cytokines such as IL-2 and IL-15, whose effects on NK cells is largely mediated by GAB3 [7]. More recently, it has become evident that, similarly to T cells, NK cells also harbor some sort of immunological memory (NKm). NK cells’ half-life is 1–2 weeks, whereas NKm cells have a half-life of 3–4 weeks [8]. NKm cells can be found in the tumor microenvironment; they secrete high levels of IFNγ, Perforin (PRF), Granulysine (GNLY), and Granzyme A and B (GZMA, GZMB) upon re-stimulation [9], and their impaired function is associated with cancer growth and dissemination [10].

NK cells kill target cells through two main mechanisms: a granule exocytosis pathway and death receptor-mediated apoptosis. Both mechanisms require the formation of an immunological synapsis between the target cell and the NK cell. In the granule exocytosis pathway, the final outcome is the degranulation of NK cells and the release of PRF, which forms pores in the plasma membrane of the target cell, allowing the entrance of GZMA and GZMB, which lead to the activation of caspase-dependent apoptosis. This cytotoxic mechanism is modulated by repressive and activating receptors, whose overall engagement (through specific membrane-bound ligands on the cancer cell) determines the final “killing” vs. “no-killing” outcome [11,12].

NK cell activating receptors include the following: MHC-I binding receptors (such as the Killer cell immunoglobulin-like receptor (KIR) family members KIR2DS1, KIR2DS3, KIR3DS5; murine Ly49 receptors; NKp80; NKG2C; and NKG2E) [13,14], receptor binding MHC-I-related sequences (NKG2D) [15], receptors binding proteins non-MHC-I-related (e.g., DNAM1 binding to CD155 and CD112) [16], Natural Cytotoxic Receptors (including NKp46, NKp44, NKp30) binding to heparan sulphate glycosaminoglycans, and other ligands excellently described in [17,18,19]. NK cell inhibiting receptors include those proteins that bind to MHC-I and proteins that have other-than-MHC-I ligands. MHC-I-specific receptors include KIR members [20,21], which are also present on the surface of some T lymphocytes, and the leukocyte immunoglobulin-like receptor (LIR) family [22]. Non-MHC-I-specific inhibitory receptors include LAG3 (whose ligand is MHC-II) [23]; TIGIT (binding the poliovirus receptor protein also known as CD155) [24]; members of the Siglec family (which bind sialic acid) [25]; and KLRG1 [26], expressed on NK and T cells whose inhibitory function is elicited by its binding to CDH1, CDH2, CDH4 (E-, N-, and R-Cadherins, respectively). Several other NK inhibitory receptors have been described, but their comprehensive list goes beyond the scope of our article.

The death receptor-mediated apoptosis consists in the expression of ligands such as FASL and TRAIL, which bind to their receptors (FAS, TRAIL-R1, TRAIL-R2) on the cancer cell, triggering FADD-mediated extrinsic caspase pathway activation, which ultimately leads to apoptosis [27].

### 2.2. Extracellular Vesicles (EVs) and microRNAs (miRs)

Extracellular vesicles are anucleated, non-self-replicative particles released by cells, whose cargo content is compartmentalized by a lipidic bilayer membrane [28]. The International Society for Extracellular Vesicles (ISEV) has published guidelines to study EVs (“MISEV2023 or Minimal information for studies of extracellular vesicles” [28]) with a clear indication of the terms to be used when describing EV nomenclature and the degree of recommendation for the usage of each specific term. According to these guidelines, the frequently previously used terms “exosomes” (to indicate EVs originating from the endosomal compartment), “microvesicles” (to indicate bigger-sized EVs budding from the plasma membrane), “ectosomes” (originating from the plasma membrane and with a wide size range) should be discouraged, unless a subcellular origin can be clearly demonstrated (limited to “exosomes” and “ectosomes”) [28]. The use of other “operational terms” such as small EVs or large EVs has a cautionary recommendation, since it is affected by the adopted separation methods [28].

Nonetheless, in reviewing the literature for the present work, we necessarily encountered the use of “traditional” terms such as “exosomes” or “microvesicles” and, in adherence with the source of the cited work, we will preserve the term utilized by the Authors, leaving the Reader to access the original publication of interest and determine the type of EVs actually studied in the specific work and according to the new guidelines, with the understanding that the Authors either published their works before the MISEV2023 guidelines were released or were less rigorous in the characterization of the EVs used in their studies.

MicroRNAs (miRs) are short single-stranded RNA oligonucleotides that do not encode for proteins and regulate mRNA translation post-transcriptionally [29]. While their gene expression regulatory functions mostly result in inhibition of translation and downregulation of the target gene-encoded protein [30], it has also been shown that miRs can increase translation of target genes [31]. Additionally, our group was the first to identify a completely novel function of miRs, as ligands of Toll-like receptor 8 (TLR8) promoting a pro-tumoral inflammatory response within the tumor microenvironment, relevant to multiple types of cancers [32,33]. In this review article, all miRs refer to the species “homo sapiens”. Therefore, the miRbase [34] nomenclature will be written without the indication of the species (e.g., “hsa-miR-186-5p” will be simply written as “miR-186-5p”).

## 3. Effects of NK-Derived Extracellular Vesicular miRs on Cancer Cells

**Figure 1:**
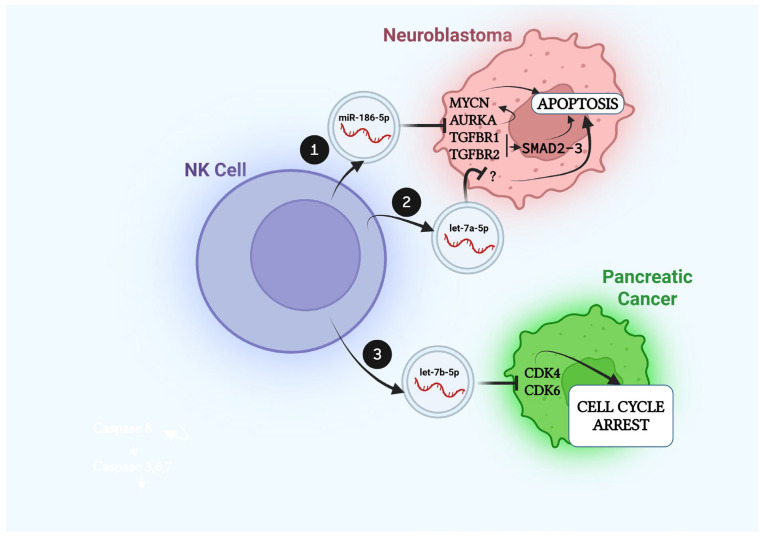
**Effects of NK cell-derived EV miRs on cancer cells.** (1). NK cells release miR-186-5p in exosomes and transfer it to neuroblastoma cells, where the miR directly targets *MYCN*, *AURKA*, *TGFBR1*, and *TGFBR2*. AURKA is a stabilizer of *MYCN*, and its targeting by miR-186-5p leads to an overall downregulation of MYCN, which induces apoptosis in neuroblastoma cells. The targeting of *TGFBR1* and *TGFBR2* blocks the SMAD2/3 signaling in neuroblastoma cells, also leading to apoptosis. (2). Biomimetic nanoparticles with NK cell-derived exosomal shell and loaded with let-7a-5p induce apoptosis of neuroblastoma cells. (3). NK cell-derived exosomes contain let-7b-5p, which induces cell cycle arrest of pancreatic cancer cell lines by directly targeting *CDK4* and *CDK6*.

NK cells are known to perform their cytotoxic effects on cancer cells through a granule exocytosis pathway and death receptor-mediated apoptosis. These two “traditional” pathways have been previously described. More recently, increasing evidence has shown that NK cells can also kill cancer cells through the release of cytotoxic EVs, and an increasing number of studies are reporting that this cytotoxicity is mediated by the miRs contained in NK-derived EVs and shuttled to cancer cells. A summary of these studies is provided in Figure 1 and Table 1.

### 3.1. NK-Derived Vesicular miRs in Neuroblastoma

Neuroblastoma (NB) is the most common pediatric solid cancer developing outside of the skull [35]. It is derived from the neural crest cells committed to differentiate into cells of the sympathetic nervous system [36]. *MYCN* is the driving oncogene for NB; however, all the efforts to silence its protein product MYCN have failed because of its structural and biochemical properties, so that MYCN is still considered “un-druggable” [37], despite more recent new scientific and technological improvements that include PROTAC/degrader technology [38]. Our group was the first to show that NK-derived EVs (characterized as exosomes) elicit a dose-dependent cytotoxic effect on NB cells and that this effect is, at least in part, due to miR-186-5p, carried as cargo by the NK-derived exosomes [39]. We showed that miR-186-5p acts as a bona fide tumor-suppressor miR in NB, since lower levels of this miR are significantly correlated with a lower event-free and overall survival in NB patients [39]. Mechanistically, we provided experimental evidence that miR-186-5p directly targets *MYCN*, *AURKA* (which encodes for MYCN-stabilizing protein Aurora Kinase A), and the genes *TGFBR1* and *TGFBR2* [39] (encoding for the receptors of Transforming Growth Factor-beta, TGFβ). The silencing of *MYCN* by miR-186-5p provides the rationale for an alternative strategy (using miRs) to achieve inhibition of this potent and un-druggable oncogene. Intriguingly, the targeting of *TGFBR1* and *TGFBR2* by miR-186-5p is able to keep the TGFβ signaling pathway silenced even in the presence of the TGFβ agonist [39]. This observation has implications on the biology of NK cells also. TGFβ is a potent inhibitor of NK cell-mediated cytotoxicity [40], and it is well known that the tumor microenvironment of several types of cancers (including NB) is imbued with TGFβ [41,42]. Therefore, by targeting the TGFβ pathway, miR-186-5p can also restore the cytotoxicity of NK cells within an immunosuppressive tumor microenvironment. An unexpected finding in our study was that NK-derived exosomes preserved their cytotoxic effects on NB cells even in conditions of starvation or in presence of TGFβ [39], both conditions significantly impairing the cytotoxicity of NK cells. This finding suggests a possible therapeutic role of NK-derived exosomes even in an unfavorable tumor microenvironment, in which NK cells fail to effectively kill cancer cells. Further studies comparing the cytotoxicity of NK cells with that of NK cell-derived exosomes are warranted. Wang G et al. were able to generate biomimetic nanoparticles composed of a hydrophilic NK cell-derived exosomal shell and loaded with -let-7a-5p [43], a widely studied miR. They showed that the combination of NK cell-derived exosomes with their biomimetic nanoparticle efficiently delivered let-7a-5p to cancer cells and exerted an anti-tumoral effect in a murine model of NB, demonstrating at the same time both the cytotoxic role of let-7a-5p in NB and the rationale for a cocktail dual therapy with NK-derived exosomes and engineered miR-enriched biomimetic nanoparticles for the treatment of NB [43]. It is important to note that the cytotoxic effects of EVs are not exclusively mediated by their miR cargo content, since it has been demonstrated that NK-derived EVs also contain PFN, GNLY, GZMA, GZMB, and other proteins well known to mediate the cytotoxic effects of NK cells on cancer cells [44].

### 3.2. NK-Derived Vesicular miRs in Pancreatic Cancer

Pancreatic cancer is a very deadly form of cancer. It is estimated that, in the U.S., the number of new cases is about 35,000 and 32,000/year in males and females, respectively, with a dismal 5 year survival rate of 13% [45]. It represents the fourth most frequent cause of cancer-related deaths in males and the third in females [45]. The first evidence that NK cell-derived exosomes elicit an anti-tumoral effect against pancreatic cancer was provided by Moretta’s lab. In an elegant series of experiments, they showed that let-7b-5p can be effectively transferred from NK cells to pancreatic cancer cells, where it inhibits cell proliferation by targeting *CDK6* (in PANC-1 cells) and *CDK4* (in MIA PaCa-2 cells) [46]. MiR let-7b-5p is part of a signature of miRs highly expressed both in NK cells and in NK cell-derived exosomes and which includes also miR-16-5p, miR-92a-3p, miR-24-3p, and miR-342-3p. Interestingly, a similar signature had been previously identified in NK cell-derived exosomes by our lab [39] and by Enomoto et al. (who profiled exosomal miRs from the NK92 cell line) [47], despite the differences in NK cell source, method of activation, and the exosomal miR high-throughput detection method employed by the different labs. These findings support the reproducibility of the NK cell-derived exosomal miR signature. Moreover, Enomoto et al. also showed that the miR signature in NK92 cell-derived exosomes is dynamic and modulated by the specific cocktail of cytokines used to stimulate NK92 cells [47]. Additionally, Moretta’s lab compared the miRNome expression in NK exosomes with its expression in NK cells, revealing that there is only partial overlapping between the two and supporting the conclusion that NK cell-derived exosomes do not fully recapitulate the miR expression of the NK cells they derive from [46]. This study also identified a “GGCUG” motif involved in the selective sorting of miRs in NK exosomes and proved that the exposure of NK cells to pancreatic cancer cells reduces the miR content in NK cell-derived exosomes and their cytotoxic properties [46], unraveling a new possible mechanism of immune escape used by pancreatic cancer cells to evade NK killing.

### 3.3. NK-Derived Vesicular Mechanisms of Cytotoxicity in Other Types of Cancers

Lugini et al. provided the first evidence that both resting and activated NK cells, derived from peripheral blood mononuclear cells (PBMCs), released exosomes containing PFN and expressing a higher-molecular-weight isoform of the membrane FASL and the typical NK cell surface markers CD56, NKp46, and NKG2D [48]. They showed that exosomes from activated NK cells were cytotoxic for Jurkat cells (T-cell leukemia) in a time- and dose-dependent manner, and their cytotoxic effect was also observed against K562 cells (chronic myelogenous leukemia) and Daudi cells (Burkitt lymphoma). Cytotoxicity was observed also against SKBR3 cells (breast cancer) and 501mel cells (metastatic melanoma); however, solid tumors were overall more resistant to NK-derived exosome-mediated cytotoxicity. Mechanistically, they observed that cytotoxicity was mediated both by the surface FASL/FAS interaction and by the release of exosomal PFN cargo. Interestingly, these Authors also showed that exosomes derived from both resting and activated NK cells from PBMCs were cytotoxic against activated but not resting immune cells [48]. Jong et al. observed a much higher cytotoxic effect of NK exosomes on breast cancer cell lines than Lugini et al., highlighting the differences in the protocol adopted to activate NK cells [44].

Melanoma B16F10 cells undergo apoptosis when treated with exosomes derived from NK-92MI cells [49]. These exosomes express CD63 and ALIX and contain PFN, FASL, and TNFα [49]. Their cytotoxic effect is time- and dose-dependent and it is at least in part mediated by the surface expression of FASL since it is significantly reduced by AF-016, a FASL inhibitor [49]. Moreover, no cytotoxicity was observed on normal cells and no side effects were observed in xenograft murine models [49]. Cytotoxicity by NK-92MI cell-derived exosomes was observed against the gastric cancer cell line SNU484 and the colorectal cancer cell line HCT-15 [49] also, suggesting that even exosomes isolated from commercially available NK cell lines (likely with no inter-donor-dependent variability) could be effectively employed as anti-cancer therapeutics in multiple types of malignancies.

## 4. Effects of Tumor-Derived Extracellular Vesicle miRs on NK Cells

**Figure 2:**
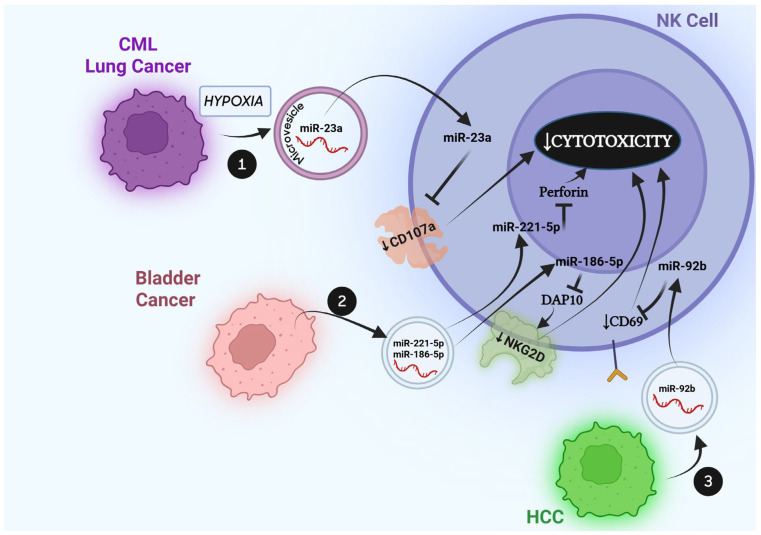
**Effects of cancer cell-derived EV miRs on NK cells.** (1). Under hypoxic conditions, chronic myelogenous leukemia and lung cancer cells release microvesicles enriched for miR-23a, which is then transferred to NK cells, where it directly targets *LAMP1*, leading to downregulation of its protein product CD107a, whose downregulation reduces NK cell cytotoxicity. (2). T24 bladder cancer cells secrete exosomes containing miR-221-5p and miR-186-5p, which are transferred to NK cells. MiR-221-5p downregulates *PFN*, reducing NK cell cytotoxicity, whereas miR-186-5p directly targets *DAP10*, whose protein product is an adapter of NKG2D, whose resulting downregulation also leads to reduced NK cell cytotoxicity. (3). Hepatocellular carcinoma-derived exosomes contain miR-92b, which is transferred to NK cells, where it directly targets *CD69*, leading to reduced NK cytotoxicity.

EVs represent a well-established means of inter-cellular communication that shapes the biology of the tumor microenvironment. While the scientific literature has identified an anti-tumoral role for NK cell-derived EVs against cancer cells, another series of studies points towards a role for tumor-derived EVs in the killing of immune cells as an effective strategy enacted by cancer cells to thrive. A summary of the studies discussed in this section is provided in Figure 2 and Table 1. In 2011, Szczepanski MJ et al. treated PBMC-derived NK cells from healthy donors, with microvesicles isolated from the plasma of patients with Acute Myeloid Leukemia (AML) [50]. They observed that AML-microvesicle-treated NK cells underwent a significant impairment of their cytotoxic activity and reduction in the expression of the activating receptor NKG2D and that this effect was mediated by the expression of TGFβ on the surface of AML-derived microvesicles, since it could be reversed by anti-TGFβ monoclonal antibodies [50]. Interestingly, they also showed that IL-15 was able to protect NK cells from the inhibitory effect caused by AML-derived microvesicles [50]. Ashiru et al., through a series of elegant experiments, showed that MICA*008, the most frequently expressed allele of *MICA*, and a ligand of NKG2D, is released by cervical cancer cells in exosomes (differently from other alleles that are shed as soluble proteins), and by binding to NKG2D on the surface of NK cells, exosomal MICA*008 induces downregulation of NKG2D from the surface of NK cells and causes a significant loss of their cytotoxic activity, representing a new strategy of exosome-mediated immune-escape mechanism [51]. Berchem et al. focused on hypoxia, a hallmark of the cancer microenvironment, and showed that, under hypoxic conditions, IGR-Heu cells (lung cancer) and K562 cells (CML) release microvesicles that are more immune-suppressive towards NK cells than the microvesicles they release under normoxic conditions [52]. They also showed that the quality of these microvesicles differs, since, under hypoxic conditions, tumors secrete microvesicles enriched for TGFβ and miR-210 and -23a [52]. They showed that miR-23a released to NK cells as the cargo of hypoxic tumor-derived EVs directly targets *LAMP1*, leading to a downregulation of its protein product CD107a (a glycosylated lysosomal membrane protein associated with NK cytotoxicity), contributing to the observed immune-suppressive effects of these microvesicles [52]. Moreover, they injected mice intradermally with normoxic or hypoxic tumor-derived microvesicles and collected NK cells from draining lymph nodes. They observed that NK cells in animals treated with hypoxic tumor-derived microvesicles displayed decreased levels of CD107a, IFNγ, and GZMB compared with NK cells from animals treated with normoxic tumor-derived microvesicles [52]. The role of tumor-derived exosomal miRs on NK cell function has been studied by Nakano et al. in a seminal paper in which they studied the impact of circulating cancer-derived exosomes on the recurrence and growth of hepatocellular carcinoma (HCC) post-transplant. They established a rat model of hepatoma and then injected naïve rats with the serum from hepatoma rats (or exosome-depleted serum from hepatoma rats as a control). In the group treated with un-depleted serum, they observed gradually increasing levels of α-fetoprotein (APF) after 4 weeks from a single injection, which remained high after 5–7 weeks [53]. They also observed increased levels of miR-92b in cancer-derived exosomes and higher expression of miR-92b in the liver of rats treated with un-depleted serum by in situ hybridization [53]. Over-expression of miR-92b in HCC cell lines increased their motility, but, more interestingly, transfection of miR-92b in NK92 cells inhibited their cytotoxicity against HCC cells at least in part by downregulating CD69, similarly to what is observed when NK92 cells are treated with HCC-derived exosomes, which are enriched for miR-92b [53]. Finally, this study observed that circulating exosomal miR-92b predicted the risk of recurrence of HCC post-transplant for HCC [53]. Bladder cancer is also able to impair NK cell cytotoxicity by releasing exosomes containing miR-221-5p and miR-186-5p [54]. Huyan et al. showed that T24 cell (bladder cancer)-derived exosomes downregulate the expression of NKG2D, NKp30, CD226, and PFN proteins in NK cells. They also experimentally proved that miR-221-5p in T24 cell-derived exosomes directly targets *PFN*, whereas exosomal miR-186-5p directly targets *DAP10*, which encodes for an essential adapter of NKG2D, contributing to its downregulation in treated NK cells [54].

**Table 1:** Summary of the studies cited in this paper.

**NK-EVs on Cancer Cells**	**Source**	**EV Cargo Content**	**Targets**	**Type of Cancer**	**Reference**
	NK from PBMCs	miR-186-5p	*MYCN*, *AURKA*, *TGFBR1*, *TGFBR2*	Neuroblastoma	[39]
	Biomimetics NPs from NK from PBMCs	let-7a-5p	N/A	Neuroblastoma	[43]
	NK from PBMCs	let-7b-5p	*CDK6*, *CDK4*	Pancreatic	[46]
	NK from PBMCs	FASL, PFN	FAS	Jurkat (T-cell leukemia), K562 (CML), Daudi (Burkitt lymphoma)	[48]
	NK from PBMCs	PFN, GNLY, GZMA, GZMB	CASP3 CASP7 CASP9	CHLA-255, CHLA-136 (neuroblastoma), SupB15, NALM6 (ALL), MCF7 (breast)	[44]
	NK-92MI	FASL	FAS	B16F10 (melanoma), SNU484 (stomach), HCT-15 (colon)	[49]
**Tumor-Derived EVs on NK Cells**	**Cancer Source of EVs**	**EV Cargo/Surface Agent**	**Targets**	**Effect**	**Reference**
	AML	TGFβ (surface)	NKG2D (downregulation)	Reduced cytotoxicity by primary NK	[50]
	HeLa (cervical cancer)	MICA*008	NKG2D	Reduced cytotoxicity by primary NK	[51]
	IGR-Heu (lung cancer), K562 (CML) under hypoxic conditions	TGFβ, miR-210, miR-23a	*CD107a* (targeted by miR-23a), IFNγ (downregulation), GZMBB (downregulation)	Reduced cytotoxicity by primary NK	[52]
	HCC	miR-92b	*CD69*	Reduced cytotoxicity by NK92 cells	[53]
	T24 (bladder cancer)	miR-221-5p miR-186-5p	NKG2D (downregulated through *DAP10* targeting by miR-186-5p), NKp30 (downregulation), CD226 (downregulation), *PFN* (downregulation by direct targeting by miR-221-5p)	Reduced cytotoxicity by primary NK	[54]

NK = Natural Killer; PBMCs = peripheral blood mononuclear cells; NPs = nanoparticles; N/A = not available; CML = chronic myelogenous leukemia; ALL = acute lymphocytic leukemia; AML = acute myeloid leukemia; IFNγ = interferon gamma; PFN = Perforin; GNLY = Granulysin; GZMA = Granzyme A; GZMB = Granzyme B; CASP3 = caspase 3; CASP7 = caspase 7; CASP9 = caspase 9; HCC = hepatocellular carcinoma.

## 5. miR-186-5p as a Key Player in NK–Cancer Cell Interaction

MiR-186-5p is clearly emerging as an interesting player in the interaction between cancer cells and NK cells, with anti-tumoral and pro-tumoral dual paradoxical functions. The anti-tumoral effects of miR-186-5p have been identified by Neviani et al. [39], who showed that miR-186-5p in EVs derived from NK cells can be effectively transferred to neuroblastoma cells in which it directly targets two driving oncogenes (*MYCN* and *AURKA*). Intriguingly, miR-186-5p has also been shown to target *TGFBR1* and *TGFBR2* [39], potentially reducing the immune-suppressive role of TGFβ on NK cell cytotoxicity. Conversely, Huyan et al. [54] showed that miR-186-5p released by bladder cancer cells in tumor-derived EVs, impairs NK cell cytotoxicity by directly targeting *DAP10*, which encodes for an adapter of NKG2D, leading to its downregulation and an overall immunosuppressive, pro-tumoral phenotype. While these contradictory findings highlight the need to further investigate the role of miR-186-5p, it is not surprising that the same miR acts both as an oncogene and as a tumor suppressor gene, as previously published [55]. MiRs are known to have a plethora of target genes, depending on the cell type, the stoichiometry of the miR:mRNA interaction, and sometimes on the species. In a previous publication, for instance, we found that the miR-15a/16-1 cluster alone is able to modulate (directly or indirectly) about 14% of the entire human genome [56]. Therefore, it is expected that, among these targeted genes, there are both oncogenes and tumor suppressor genes. What affects the overall outcome, depends again on the gene expression profile in a specific cell type and likely the concentration of miR in reference to its targeted mRNAs. While we can only speculate that these are some of the reasons why miR-186-5p showed both pro- and anti-tumoral effects in the NK–cancer cell interaction, there is no doubt that its role is prominent in this interaction and that it deserves further studies to better understand this fascinating miR biology.

## 6. Conclusions and Future Directions

An increasing number of studies focus on the anti-cancer properties of NK-derived EVs and point towards a role for tumor-derived EVs as an important mechanism of immune escape. There seems to be a consensus on the signature of miRs contained in NK-derived EVs, and an increasing number of publications are unraveling the molecular mechanisms mediating their cytotoxic effects. Conversely, different cancer cells seem to mediate their inhibitory effect on NK cell function through the release of different types of vesicular miRs. Nonetheless, the underlying molecular mechanisms responsible for the impaired NK cell function seem to converge on the downregulation of NK cell activating receptors and the perturbation of the TGFβ pathway. It is important to mention that Enomoto et al. have focused on the effects of cytokines on the anti-tumoral activity of NK-derived exosomes. They found that the combination of IL-15 and IL-21 (more than the single cytokines) increases the cytotoxicity of NK-derived exosomes against multiple cancer cell lines (K652, Jurkat, A549, HeLa), and that effect is in part mediated by the upregulation of CD226 on NK-EVs and by the increased concentration of their cargo of miRs with a potential cytotoxic role [47]. They also proved that NK-EVs are uptaken by cancer cells through macropinocytosis [47]. It is worth noting that the miR signature of cytokine-treated NK92-derived EVs is remarkably consistent with that in two other studies [39,57], supporting the reproducibility and consistency of the NK-EV miRNome across different NK cells sources (primary vs. NK-92). In their work, Dosil et al. investigated the effects of NK-EVs on T-cell anti-cancer responses and identified a signature of NK vesicular miRs (namely, miR-10b-5p, -92a-3p, and -155-5p) and isomiRs (miR isoforms that differ from the canonical miR sequence due to variations such as 5′- or 3′-end modifications, nucleotide additions/substitutions) promoting Th1 polarization and IFNγ and IL-2 production [57]. Mechanistically, they showed that miR-10b-5p and -92a-3p in NK-EVs induce downregulation of GATA3 and upregulation of TBX21 in CD4+ T cells [57]. Moreover, they observed that NK-EVs increase the presentation and co-stimulatory properties of dendritic cells by upregulating MHCII and CD86 [57]. The study also showed a higher number of isomiRs (with post-transcriptional modifications such as adenylation and cytosylation) in NK-EVs than in the NK cells of origin and identified RNPA2B1 consensus sequences “GGCAGU” and “UGGA” in miRs packed into NK-EVs, supporting an active machinery involved in the sorting of exosomal miRs [57]. While still in the early stages, these fundamental discoveries highlight a broader role for NK-EVs, which encompasses all cellular components of the tumor microenvironment, further corroborating their central function in orchestrating the biology of the tumor microenvironment. While further studies are warranted to better understand the role of NK vesicular miRs on cancer cells and the impact of tumor-derived vesicular miRs on NK function, new strategies are being implemented to selectively and specifically enrich NK-derived EVs for the miRs with cytotoxic properties. This will achieve the dual goal of increasing the cytotoxic properties of NK-EVs while compartmentalizing the miRs of interest and ensuring a better on-target delivery while reducing the risks of their extra-vesicular degradation. We foresee these efforts as one of the most promising future directions in this field. Clearly, the generation of artificial nanoparticles enriched for cytotoxic miRs is another attractive and pursued strategy. However, miRs are likely not the only cargo component responsible for the anti-cancer effects of NK-EVs. Additionally, surface proteins in NK-EVs have been clearly shown to be involved in the observed cytotoxicity. If we also consider the surface expression of Fc receptors on NK-EVs, it is conceivable that antibodies can be used against tumor-associated antigens (TAAs) to drive the tropism of NK-EVs, further achieving a specific cytotoxic effect with reduced risk of side effects. From a practical translational perspective, the use of NK-EVs in the clinical setting requires manufacturing processes and quality-control (QC) testing that comply with current good manufacturing practice (GMP). While this is not yet currently implemented, we envision a path that recapitulates the successful stories of GMP-grade EVs derived from other cells and currently tested in clinical trials. At the time of this review article, using ClinicalTrials.gov as a source and searching with the words “extracellular vesicles” under “Intervention/treatment”, there are 179 clinical trials (105 Interventional, 71 Observational, 3 Expanded access study types) testing EVs in different conditions, spanning from skin ulcers to inflammatory bowel diseases, to bone diseases, to cardiology. In cardiology, for example, cardiovascular progenitor cell (CPC)-derived EVs are currently being tested in a phase-I clinical trial in severely symptomatic patients with drug-refractory left ventricular (LV) dysfunction secondary to non-ischemic dilated cardiomyopathy (NCT05774509) [58]. In patients with relapsed/refractory Acute B-Cell Leukemia, early phase I clinical trial NCT06890494 is testing the safety and efficacy of EVs derived from mesenchymal stem cells (MSCs) expressing Bi-specific T-cell Engagers (BiTEs) against CD3 and CD19. Currently no ongoing clinical trial is testing NK-EVs. Obstacles to their clinical use include the need for more studies on their pharmacokinetics, biodistribution, safety, and regulatory pathways. When injected in vivo (primary in the tail vein of mice), NK-EVs accumulate primarily in the liver, spleen, and lungs (consistently with what is observed for many other types of EVs) [59]. NK-EV half-life has not been clearly defined in preclinical settings, and donor variability seems to affect the physicochemical properties of NK-EVs such as size distribution, surface protein profile, glycosylation patterns, lipid composition, protein, and miR cargo loads, ultimately affecting their pharmacokinetics and biodistribution properties [60]. Another area that deserves more investigation is the combination of NK-EVs with existing chemo/immune/hormone-therapy regimens. Recently, a study showed that NK-EVs can be successfully collected from patient-derived NK cells and used to enhance cytotoxicity and immune cell recruitment in organoids from primary non-small-cell lung cancer cells in combination with nivolumab [61]. Combining NK-EVs with TGFβ inhibitors seems a logical step to improve anti-cancer efficacy, and it has been successfully tested in preclinical models [62]. While CAR-NK cell-derived EVs can be enriched for cytotoxic miRs to improve cancer cell killing [63], a direct combination of CAR-based therapies and NK-EVs remains an exciting area for future preclinical investigation. In conclusion, the clinical use of NK-EVs as cancer therapeutics is still in its infancy, but mirroring the successful stories of EVs derived from other cells and currently used in clinical trials and addressing the areas in need of further preclinical investigation will ultimately pave the road towards clinical trials with NK-EVs (alone or combined to other treatments) and will likely impact on the survival of cancer patients.
